# Effects of Beet‐Vinasse (Condensed Molasses Soluble) Addition on the Performance, Milk Fatty Acid Profile, Digestibility, Blood Parameters, and Ruminal Fermentation of Early Lactating Holstein Cows

**DOI:** 10.1002/vms3.70990

**Published:** 2026-05-14

**Authors:** A. Rahimi, F. Fatehi, H. Rafiee, A. Kahyani

**Affiliations:** ^1^ Department of Animal Sciences, College of Agriculture Isfahan University of Technology Isfahan Iran; ^2^ Department of Animal Science, College of Agriculture and Natural Resources University of Tehran Karaj Iran; ^3^ Animal Science Research Department, Isfahan Agriculture and Natural Resources Research and Education Center Agricultural Research, Education and Extension Organization (AREEO) Isfahan Iran; ^4^ Department of Animal Science, School of Agriculture Shiraz University Shiraz Iran

**Keywords:** by‐product, dairy cow, milk fatty acid profile, nutrient digestibility, vinasse

## Abstract

**Background:**

Beet vinasse (VIN), a by‐product of sugar beet processing, is rich in non‐protein nitrogen (NPN), potassium, and prebiotic compounds, offering potential as a feed additive to enhance dairy cow performance while reducing environmental waste.

**Objectives:**

This study investigates the effects of VIN supplementation at 0%, 1.5% (300 g/day) or 3% (600 g/day) on feed intake, feed efficiency (FE), milk production and composition, apparent nutrient digestibility, blood metabolites and ruminal fermentation in early‐lactation Holstein dairy cows.

**Methods:**

Twenty‐four Holstein dairy cows (15 ± 3 days in milk, 30 ± 2.9 kg of milk/day) were used in a completely randomized design with three treatments: (1) control diet without beet VIN supplementation (0 g VIN/cow/day, 0% VIN; V0); (2) diet supplemented with 300 g VIN/cow/day (1.5% VIN; V1.5); and (3) diet supplemented with 600 g VIN/cow/day (3% VIN; V3). VIN was top‐dressed over the total mixed ration. The experiment included a 15‐day adaptation period and a 30‐day sampling period.

**Results:**

Dry matter intake (DMI) was unaffected by treatments, whereas FE and milk yield increased linearly with increasing VIN levels. Fat‐corrected milk (FCM), energy‐corrected milk (ECM) and yields of milk fat, true protein, lactose and solids non‐fat (SNF) increased linearly from V0 to V3. Milk fat percentage exhibited a quadratic response, with highest milk fat observed at the V1.5 level. True protein and concentrations of de novo and saturated fatty acids increased linearly with VIN inclusion. Apparent digestibility of dry matter, organic matter and crude protein improved linearly with increasing VIN levels. Ruminal pH, acetate proportion and acetate to propionate ratio increased linearly, whereas the proportions of valerate and isovalerate decreased linearly. Plasma globulin, total protein and blood urea nitrogen concentrations increased linearly, whereas plasma glucose concentration decreased linearly with increasing VIN.

**Conclusions:**

VIN supplementation at 1.5% or 3% enhances milk production, FE and nutrient digestibility in early‐lactating cows without affecting DMI. However, increased plasma globulin may suggest a mild inflammatory response, necessitating further investigation.

**Implications:**

VIN supplementation at 1.5%–3% enhances nutrient digestibility, ruminal fermentation and production efficiency in early‐lactating Holstein cows, likely due to its NPN and mineral content. However, without rumen microbial or immune response data, long‐term safety and efficacy require further study. VIN offers a cost‐effective, environmentally sustainable feed additive pending validation.

## Graphical abstract

Vinasse supplementation at 1.5%–3% enhances nutrient digestibility, ruminal fermentation and production efficiency in early‐lactating Holstein cows. However, without rumen microbial or immune response data, long‐term safety and efficacy require further study.

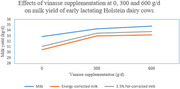

## 1 Introduction

In recent years, there has been a growing interest in utilizing by‐products from the food industry as alternative animal feeds, primarily due to environmental concerns and rising feed costs. Feeding these by‐products and food wastes can mitigate the environmental impacts of the food industry while improving its profitability and productivity (Zali et al. 2017). Additionally, increasing global population growth has escalated the demand for animal products, intensifying the need for sustainable and efficient feed resources (Webb and Erasmus 2013). Due to shortages in traditional feedstuffs, it is crucial to explore alternative by‐products that can partially replace conventional nutrients in animals (Maneerat et al. 2015).

Vinasse (VIN) is a by‐product derived from molasses after fermentation processes that produce ethanol, citric acid, yeast and other substances involved in sugar production. The fermentation process entails the removal and conversion of sugars (Stemme et al. 2005). Due to the extensive consumption of molasses sugars by yeasts during alcohol production, VIN typically contains low sugar levels (Carrilho et al. 2016). However, it is rich in crude protein (CP) (predominantly non‐protein nitrogen [NPN] in the forms of aspartic acid, glutamic acid and betaine), ash (notably potassium) and B‐complex vitamins (Stemme et al. 2005). The fibre and fat content is minimal and often negligible. Furthermore, VIN exhibits high glycerol content and probiotic properties (Weigand and Kirchgessner 1980; Fernández et al. 2009; Yalcin et al. 2010). Given its large production volume, effective disposal methods of VIN are crucial to prevent environmental contamination.

Due to high potassium, sulphates and NPN content, along with probiotic properties, VIN can serve as a valuable ingredient in animal feed formulations as a source of nutrients and minerals (Stemme et al. 2005; Lopez‐Campos et al. 2011). Research indicates that incorporating VIN into the diet of fattening sheep reduces dry matter intake (DMI) and growth without affecting carcass characteristics (Lopez‐Campos et al. 2011). Similarly, a mixture of bagasse and VIN fed to steers decreased DMI without any change in body weight (Maneerat et al. 2015). Meanwhile, the inclusion of 5% VIN in growing calves’ rations showed no adverse effects on growth while promoting improved carcass composition (Zali et al. 2017(. Moreover, Zali et al. (2019) reported that VIN can be included in goat kids’ diets up to 12% without any adverse effects on performance but with potential of reductions in the internal fat content. In lactating cows fed varying levels of condensed molasses fermentation solubles (CMS), daily milk yield and DMI decreased when CMS exceeded 3% of the diet (Ma et al. 2021). Additionally, Rahimi et al. (2021) found that a supplementation of 5% dry matter VIN (1.1 kg DM/day) improved nutrient digestibility (dry matter [DM] and neutral detergent fibre [NDF]) while reducing feed costs without negatively impacting DMI in mid‐lactation dairy cows. The inconsistency in the effects of VIN‐molasses on ruminant performance necessitates further investigation.

Early lactation represents a physiologically and metabolically distinct period marked by heightened nutrient demands and health challenges. Previous investigations have primarily focused on mid‐lactation cows, leaving a critical knowledge gap regarding VIN supplementation during early lactation. Specifically, Rahimi et al. (2021) investigated cows at 155 ± 25 days in milk (DIM) with a milk yield of 32 ± 3 kg/day, whereas Ma et al. (2021) used cows at 55 ± 5 DIM, producing 35 ± 2.5 kg/day. This gap is significant due to the unique metabolic stress and nutrient requirements in the first 30 days of lactation, which considerably impact production and health outcomes (Martens 2023). Therefore, evaluating the effects of VIN during early lactation could provide valuable insights for nutritional management strategies aimed at improving performance and welfare.

To our knowledge, limited information is available regarding the influence of VIN on the performance, milk fatty acid (FA) composition, rumen fermentation, digestibility and blood metabolites in early lactating cows. We hypothesize that including VIN in the diet could enhance performance without adversely affecting the DMI of dairy cows during early lactation. Thus, the current study aimed to evaluate the effects of incorporating VIN at different levels as a feed additive on DMI, milk production and FA composition, rumen fermentation, nutrient digestibility and selected blood parameters in early lactating dairy cows.

## 2 Materials and Methods

All experimental procedures adhered to the Animal Care Committee of Isfahan University of Technology by the Iranian Council of Animal Care (1995) with approval number (IRN‐0002‐5‐16). The experiment was performed from July to September 2020 at a dairy farm in Karaj, Iran. To ensure cow welfare, animals were housed in individual tie‐stalls with constant access to clean water and comfortable bedding. Daily veterinary oversight monitored cows for signs of distress during feeding, milking and sampling. Rumen fluid and blood sampling were performed by trained personnel following established protocols to minimize stress (Geishauser 1993).

### 2.1 Animals and Treatments

A complete randomized design was employed in this study, which involved 24 first‐lactation Holstein dairy cows. At the start, cows averaged 15 ± 3 DIM, 30 ± 2.9 kg of milk per day and 620 ± 44 kg of body weight. To ensure balance, the cows were categorized into eight groups on the basis of their actual milk yield and calving date. They were individually housed in pens measuring 4 × 4 m^2^, featuring regularly cleaned concrete floors. The cows had ad libitum access to a total mixed ration (TMR) and water. The dietary treatments consisted of the following: V0: A diet containing 0% VIN. V1.5: A diet containing 1.5% VIN, equivalent to 300 g per day per cow. V3: A diet containing 3% VIN, equivalent to 600 g per day per cow.

The TMR was freshly prepared on a daily basis and offered twice daily at 0700 and 1500 in equal amounts, with the aim of ensuring 5%–10% feed refusals. The ingredients and chemical composition of the basal diet are provided in Table 1. The diet was formulated to meet or exceed the nutrient requirements by the National Research Council (2001). VIN utilized in the study was sourced from the Bidestan Alcohol and Foodstuff Production Company (Qazvin, Iran). The chemical composition of VIN is outlined in Table 2. VIN was incorporated into the TMR, based on an as‐fed basis, and it was top‐dressed twice daily by mixing it into the upper portion of the TMR during each feeding. The entire trial lasted for 45 days, which included a 15‐day adaptation period and a subsequent 30‐day period for sampling and data collection.

**TABLE 1:** Ingredients and chemical composition of the experimental diet.

Items	% On dry matter basis
Ingredients	
Alfalfa hay	16.81
Corn silage	22.40
Corn grain	15.2
Barley grain	16.8
Soybean meal	9.00
Beet pulp	4.65
Wheat bran	1.70
Meat meal	5.03
Cottonseed	2.90
Corn gluten meal	0.86
Fat powder^a^	1.90
Calcium carbonate	0.30
Sodium bicarbonate	1.00
Dicalcium phosphate	0.19
Bentonite	0.26
Toxin binder	0.07
Magnesium oxide	0.26
Vitamin‐mineral premix^b^	0.56
Salt	0.11
Chemical composition	
Dry matter	52
Crude protein	16.7
Ether extract	4.90
Ash	7.85
Neutral detergent fibre	32.7
Non‐fibre carbohydrates^c^	37.8
Calcium^d^	1.36
Phosphorus^d^	0.64
Net energy for lactation^d^ (Mcal/kg)	1.65

^a^ Palmac 80‐16, Erafeed, Malaysia. Composition: moisture 0.5%, crude fat 99.5% (C14:0, 3%; C16:0, 85%; C18:0, 2%; C18:1, 8%–10%; C18:2, 2%).

^b^ Premix contained: kg of mineral‐protein supplement containing 1,600,000 IU of vitamin A, 250,000 IU of vitamin D, 7000 IU of vitamin E, 250 mg of biotin, 3 g of monensin, 16% calcium, 4% phosphor, 3% sulphur, 5% magnesium, 10 g of manganese, 14 g of zinc, 1 g of iron, 6 g of copper, 100 mg of selenium, 200 mg of iodine and 80 mg of cobalt.

^c^ Nonfibrous carbohydrates: 100 − (CP + NDF + ether extract + ash) (NRC 2001).

^d^ Calculated from NRC (2001).

**TABLE 2:** Chemical composition of vinasse.

Item	% Of dry matter
Dry matter	50.5
Crude protein	27.8
Ash	23.2
Calcium	2.98
Phosphorous	0.8
Sodium	3.11
Potassium	8.8
Chloride	3.2
Glycerol	3.35
Betaine	3.73

### 2.2 Sampling, Measurements and Laboratory Analyses

During the sampling period, the quantities of TMR offered to each cow were weighed on a daily basis. Additionally, the leftovers (orts) were recorded every morning before the cows were fed. The TMR and its samples were collected and subsequently dried at 68°C for 48 h in a forced‐air oven to facilitate the calculation of DMI. Feed intake was determined by subtracting the total amount of TMR offered daily from the amount of refusals. Furthermore, the dried TMR samples were ground to pass through a 1‐mm screen in a mill (Ogaw Seiki CO. Ltd., located in Tokyo, Japan). These ground samples were then subjected to analysis according to the methods outlined by AOAC (2002) for DM (method 925.40), CP (method 2001.11), ether extract (EE, method 920.39) and ash (method 942.05). The NDF content was determined following the procedures described by Van Soest et al. (1991), which involved the use of heat‐stable α‐amylase.

On Day 38 of the study, rumen fluid samples were obtained approximately 4 h after the morning feed. The collection of rumen contents was carried out by utilizing a custom‐designed stomach pump and a tube inserted through the rumen into a 500 mL container, following the method described by Geishauser (1993). The initial 200 mL of fluid pumped out was discarded, and the subsequent portion was filtered through four layers of cheesecloth. Immediately after filtration, the pH of the filtered rumen fluid was measured using a portable pH meter (HI 8318; Hanna Instruments, Cluj‐Napoca, Romania). Subsequently, two subsamples of the ruminal fluid were taken and mixed with chilled 25% (wt/vol) metaphosphoric acid (H_3_PO_4_). These samples were then stored at −20°C for later analysis of volatile FAs (VFA) and NH_3_‐N. Upon thawing, the concentration of NH_3_‐N was determined using the colorimetric phenol‐hypochlorite method outlined by Broderick and Kang (1980). For VFA analysis, another subsample of the rumen fluid was thawed and centrifuged at 10,000× *g* at 4°C for 20 min. The VFA content was analysed using gas chromatography with a 0.25 × 0.32, 0.3 µm i.d. fused silica capillary (model no. CP‐9002 Vulcanusweg 259 a.m., Chrompack, Delft, the Netherlands), following the procedure described by Bal et al. (2000).

On Days 21 and 41 of the study, blood samples were collected from the coccygeal vein. Heparinized tubes (7 mL per sample; Vacutainer, Becton Dickinson, Franklin Lakes, NJ, USA) were used for blood collection, approximately 3 h after the morning feeding. Immediately after collection, the tubes were placed in iced water to maintain sample integrity. Subsequently, the blood samples were centrifuged at 3000 rpm for 15 min. The resulting plasma was carefully separated and transferred into 1.5‐mL microtubes and stored at −20°C until further analysis. After the experimental period, the plasma samples were thawed, and the concentrations of various components were determined using an automatic analyser (Alcyon‐300 Auto analyser; DRG Instruments GmbH, Marburg, Germany) and commercially available kits (Pars Azmoon, Tehran, Iran). The analytes measured included glucose (Kit no. 93008), total protein (Kit no. 9304), cholesterol (Kit no. 2118), albumin (Kit no. 9307) and triglyceride (Kit no. 2109). The determination of serum globulin was performed by subtracting the albumin value from the total plasma protein.

Milk yield was recorded thrice daily (0700, 1500 and 2300 h) during the 30‐day data collection period and averaged daily. Milk samples were collected weekly during data collection period at each milking (three times daily). For each cow, daily composite samples were prepared by pooling equal volumes of milk from the three milking within the same day. Samples were preserved with potassium dichromate and stored at 4°C. Samples were analysed for fat, true protein, lactose, solids‐not‐fat (SNF), milk urea nitrogen (MUN), non‐esterified FAs (NEFA), beta‐hydroxybutyrate (BHBA) and FAs using Fourier‐transform mid‐infrared spectroscopy (FTIR, CombiScope FTIR 600 HP, Delta Instruments, Drachten, the Netherlands). FA profiles were analysed individually for each cow. The milk composition for each day was calculated on the basis of the composite of each milking, which was corrected for milk yield.

Yields of 3.5% fat‐corrected milk (FCM, kg/day) and energy‐corrected milk (ECM, kg/day) were calculated according to the following equations as described by Dairy Records Management Systems (2014):

3.5% FCM = (0.432 × kg of milk yield) + (16.218 × kg of fat yield).

ECM = (12.95 × kg of fat yield) + (7.2 × kg of protein yield) + (0.327 × kg of milk yield).

Feed efficiency (FE) was calculated as actual and component‐corrected milk yield (kg/day) divided by DMI (kg/day).

Faecal subsamples were taken per cow (the last 4 days of the period) in the same quantity (300 g) at 9‐h intervals at 1700, 0200, 1100, 2000, 0500, 1400, 2300 and 0800 h. Faeces samples were taken when cows defecated or by rectal grab sampling and were subsequently pooled within cows in buckets and stored frozen at −20°C. Faecal samples were thawed and then dried at 60°C for 72 h for DM determination and chemical analysis. DM (method ID 934.01), ash (method ID 942.05), EE (method ID 920.30) and CP (method ID 984.13) of faeces were determined by the procedures of AOAC (2002). NDF (using heat‐stable alpha‐amylase, 100 µL/sample) and acid detergent fibre (ADF), according to Van Soest et al. (1991) with the ANKOM Fiber Analyzer system (ANKOM Technology, Macedon, NY, United States). The acid‐insoluble ash contents of the both feed and faeces samples were used as an internal marker for determining the apparent total tract digestibility of nutrients, as described by Van Keulen and Young (1977).

### 2.3 Statistical Analysis

The data were analysed using a completely randomized design in the MIXED procedure of SAS (V 9.4). The statistical model included fixed effects of treatment and time, a random effect of cow nested within treatment, and a repeated statement for time (day) to account for within‐subject correlations. Treatments were balanced on the basis of actual milk yield and calving date. The model is expressed as

*Y_ijk_* = *µ* + *T_i_* + *D_j_ *+ (*TD*)*_ij_* + *C*(*T*)*_ik_* + *E_ijk_* where *Y_ijk_* represents the dependent variable, *T_i_* is the fixed effect of treatment (*i* = 1, 2 and 3), *D_j_* is a categorical fixed effect to capture potential non‐linear effects of time across the repeated measures (*j* = 1, 2, 3, … and 30), (*TD*)*_ij_* represents the interaction effect between treatment and time, *C*(*T*)*_ik_* is the random effect of cow within treatment, and *E_ijk_* is the residual error. Data were tested for normality using the PROC UNIVARIATE procedure in SAS by examining skewness, kurtosis and the Shapiro–Wilk test. Moreover, as part of model validation, variance inflation factors (VIFs) were calculated to assess multicollinearity among fixed effects; all VIFs were within acceptable ranges and thus not reported. For variables that did not meet normality assumptions, the PROC GLIMMIX procedure was used, assuming a Poisson distribution with a logarithmic link function. Model fit was improved by making necessary adjustments and selecting appropriate distributions. To account for repeated measures, various covariance structures—including Huynh–Feldt (HF), compound symmetry (CS), Toeplitz (TOEP) and first‐order autoregressive (AR(1))—were evaluated. The structure with the lowest corrected Akaike's information criterion (AIC) was selected.

Additionally, linear and quadratic contrasts were conducted to assess specific treatment comparisons. Statistical significance was declared at a significance level of *p* < 0.05, and trends were considered at *p* < 0.10.

## 3 Results

As shown in Table 3, DMI was unaffected by dietary treatments. Milk yield (*p* < 0.01), FCM (*p* < 0.01), ECM (*p* < 0.01) and FE (*p* < 0.01) increased linearly with increasing VIN levels in the diets, with milk yield rising from 32.9 kg/day in V0 to 34.3 kg/day in V1.5 and 34.8 kg/day in V3. Yields of milk fat (*p* < 0.01), true protein (*p* < 0.01), lactose (*p* = 0.02) and SNF (*p* = 0.01) also increased linearly in cows fed increasing amounts of VIN. Milk fat concentration responded quadratically to VIN supplementation (*p* = 0.04), with the highest value in V1.5 (3.38%) compared with V0 (3.15%) and V3 (3.29%). True protein concentration increased linearly (*p* = 0.03) from 2.63% in V0 to 2.78% in V1.5 and 2.74% in V3. No effects were observed on lactose concentration, SNF concentration or MUN.

**TABLE 3:** Effects of dietary supplementation of vinasse at either 300 g/day (V1.5), 600 g/day (V3) or no supplementation (V0) on dry matter intake, feed efficiency and milk yield and composition of early lactating Holstein dairy cows (*n* = 8 per treatment).

Items	Treatments			SEM	*p* value	
	V0	V1.5	V3		Linear	Quadratic
Dry matter intake (kg/day)	20.4	20.2	20.0	0.66	0.50	0.99
Yield (kg/day)						
Milk	32.9^b^	34.3^a^	34.8^a^	0.41	<0.01	0.41
Energy‐corrected milk	30.5^b^	33.0^a^	33.2^a^	0.55	<0.01	0.09
3.5% Fat‐corrected milk	31.1^b^	33.5^a^	33.8^a^	0.58	<0.01	0.11
Fat	1.03^b^	1.15^a^	1.14^a^	0.02	<0.01	0.06
True protein	0.87^b^	0.957^a^	0.96^a^	0.01	<0.01	0.07
Lactose	1.48^b^	1.53^ab^	1.57^a^	0.02	0.02	0.97
Solids not‐fat	2.69^b^	2.79^ab^	2.87^a^	0.04	0.01	0.80
Milk composition (%)						
Fat	3.15^b^	3.38^a^	3.29^ab^	0.06	0.12	0.04
True protein	2.63^b^	2.78^a^	2.74^a^	0.03	0.03	0.05
Lactose	4.51	4.47	4.51	0.03	0.99	0.32
Solids not‐fat	8.20	8.18	8.24	0.05	0.59	0.53
MUN (mg/dL)	12.56	12.43	12.48	0.36	0.87	0.84
Feed efficiency (kg/kg)	1.66^b^	1.70^ab^	1.74^a^	0.02	<0.01	0.99

*Note*: Means within a row with different superscripts (a,b) are significantly different (*p *< 0.05).

Abbreviation: MUN, milk urea nitrogen.

The milk FA profile was selectively altered by VIN supplementation (Table 4). Proportions of de novo (*p* = 0.01) and saturated (*p* < 0.01) FAs increased linearly with increasing VIN in the diets, from 23.8% and 51.2% in V0 to 25.0% and 64.7% in V3, respectively. No treatment effects were observed on mixed, preformed, unsaturated, monounsaturated or polyunsaturated FAs, nor on individual C16:0, C18:0 or C18:1 *cis*‐9 proportions. Milk NEFA and BHBA concentrations remained unaffected.

**TABLE 4:** Effects of dietary supplementation of vinasse at either 300 g/day (V1.5), 600 g/day (V3) or no supplementation (V0) on fatty acids profile (g/100 g total fatty acids) in milk of early lactating Holstein dairy cows (*n* = 8 per treatment).

Items	Treatments			SEM	*p* value	
	V0	V1.5	V3		Linear	Quadratic
De novo fatty acids^a^	23.8^b^	24.7^a^ ^b^	25.0^a^	0.33	0.01	0.58
Mixed fatty acids^a^	35.6	35.4	35.3	0.40	0.61	0.86
Preformed fatty acids^a^	40.6	40.1	39.7	0.48	0.20	0.80
Saturated fatty acids	51.188^b^	63.214^a^ ^b^	64.733^a^	3.994	0.001	0.935
Unsaturated fatty acids	32.863	31.533	30.054	0.835	0.621	0.283
Mono unsaturated fatty acids	26.166	27.995	26.153	0.632	0.412	0.381
Poly unsaturated fatty acids	3.958	3.883	3.585	0.176	0.606	0.721
C:16	35.3	35.840	33.3	0.63	0.63	0.95
C:18	9.54	9.84	8.08	0.81	0.81	0.88
C18:1 *cis*‐9	22.3	21.65	19.50	0.85	0.20	0.61
Non esterified fatty acids (µEq/L)	457.26	436.16	424.89	20.358	0.259	0.841
BHBA (mmol/L)	0.094	0.095	0.102	0.005	0.287	0.604

*Note*: Means within a row with different superscripts (a,b) are significantly different (*p *< 0.05).

^a^ De novo FA are defined as FA with chain length <16 carbons (originated from mammary synthesis), preformed FA are defined as FA with chain length >16 carbons (originated from blood) and mixed FA (originated from both sources, C16:0 plus C16:1 *cis*‐9 and *iso*‐C16:0).

Apparent total‐tract digestibility data are presented in Table 5. Digestibility of DM (*p* < 0.01), OM (*p* < 0.01) and CP (*p* < 0.01) increased linearly with increasing VIN levels, from 66.1%, 69.0% and 66.2% in V0 to 71.3%, 74.4% and 72.8% in V3, respectively. Digestibility of NDF, ADF and EE was not affected by dietary treatments.

**TABLE 5:** Effects of dietary supplementation of vinasse at either 300 g/day (V1.5), 600 g/day (V3) or no supplementation (V0) on digestibility (%) of early lactating Holstein dairy cows (*n* = 8 per treatment).

Items	Treatments			SEM	*p* value	
	V0	V1.5	V3		Linear	Quadratic
Dry matter	66.1^b^	69.8^a^	71.3^a^	1.24	<0.01	0.47
Organic matter	69.00	72.82	74.39	1.35	<0.01	0.49
Crud protein	66.2^b^	73.3^a^	72.8^a^	1.80	<0.01	0.10
Neutral detergent fibre	50.8	53.3	54.3	1.59	0.14	0.69
Acid detergent fibre	45.6	46.4	47.4	1.10	0.26	0.93
Ether extract	69.7	71.9	71.6	1.17	0.26	0.37

*Note*: Means within a row with different superscripts (a,b) are significantly different (*p *< 0.05).

Rumen fermentation parameters are indicated in Table 6. Ruminal pH (*p* < 0.01), acetate proportion (*p* < 0.01) and acetate‐to‐propionate ratio (*p* = 0.02) increased linearly as VIN increased in the diets, from 6.03, 49.9 mol/100 mol, and 2.02 in V0 to 6.53, 55.6 mol/100 mol, and 2.61 in V3, respectively. Proportions of valerate (*p* < 0.01) and isovalerate (*p* = 0.04) decreased linearly from 1.11 and 4.20 mol/100 mol in V0 to 0.76 and 3.32 mol/100 mol in V3. Total VFA, propionate, butyrate and NH_3_‐N concentrations were unaffected.

**TABLE 6:** Effects of dietary supplementation of vinasse at either 300 g/day (V1.5), 600 g/day (V3) or no supplementation (V0) on rumen fermentation parameters of early lactating Holstein dairy cows (*n* = 8 per treatment).

Items	Treatments			SEM	*p* value	
	V0	V1.5	V3		Linear	Quadratic
pH	6.03^c^	6.28^b^	6.53^a^	0.07	<0.01	0.95
Total volatile fatty acids (mM)	98.7	97.8	93.2	6.31	0.54	0.80
Acetate (mol/100 mol)	49.9^b^	52.9^ab^	55.6^a^	1.48	<0.01	0.93
Propionate (mol/100 mol)	25.3	24.0	22.2	1.27	0.10	0.89
Butyrate (mol/100 mol)	19.5	18.5	18.1	0.88	0.27	0.81
Isovalerate (mol/100 mol)	4.20^a^	3.56^ab^	3.32^b^	0.28	0.04	0.57
Valerate (mol/100 mol)	1.11^a^	0.98^ab^	0.76^b^	0.08	<0.01	0.67
Acetate/Propionate ratio	2.02^b^	2.26^ab^	2.61^a^	0.17	0.02	0.78
NH_3_‐N (mg/dL)	11.2	11.8	11.7	0.94	0.72	0.73

*Note*: Means within a row with different superscripts (a,b) are significantly different (*p *< 0.05).

Plasma metabolite concentrations are shown in Table 7. Glucose concentration decreased linearly (*p* < 0.01) from 52.6 mg/dL in V0 to 46.6 mg/dL in V3. In contrast, plasma globulin (*p* = 0.02), total protein (*p* = 0.01) and BUN (*p* = 0.01) concentrations increased linearly from 3.22 g/dL, 6.47 g/dL and 14.6 mg/dL in V0 to 3.58 g/dL, 6.81 g/dL and 15.3 mg/dL in V3, respectively. No effects were observed on cholesterol, triglyceride, albumin or albumin‐to‐globulin ratio.

**TABLE 7:** Effects of dietary supplementation of vinasse at either 300 g/day (V1.5), 600 g/day (V3) or no supplementation (V0) on plasma parameters of early lactating Holstein dairy cows (*n* = 8 per treatment).

Items	Treatments			SEM	*p* value	
	V0	V1.5	V3		Linear	Quadratic
Glucose (mg/dL)	52.6^a^	50.9^a^	46.6^b^	1.46	<0.01	0.45
Cholesterol (mg/dL)	159	162	165	9.73	0.62	0.97
Triglyceride (mg/dL)	16.7	19.4	18.5	2.01	0.52	0.49
Albumin (g/dL)	3.15	3.13	3.00	0.07	0.18	0.58
Globulin (g/dL)	3.22^b^	3.40^b^	3.58^a^	0.11	0.02	0.99
Albumin/Globulin ratio	3.25	3.15	3.23	0.05	0.80	0.23
Total protein (g/dL)	6.47^b^	6.56^ab^	6.81^a^	0.09	0.01	0.43
Blood urea nitrogen (mg/dL)	14.6^b^	14.8^ab^	15.3^a^	0.20	0.01	0.79

*Note*: Means within a row with different superscripts (a,b) are significantly different (*p *< 0.05).

## 4 Discussion

The absence of an effect of VIN supplementation at 1.5% (300 g/day) or 3% (600 g/day) on DMI (Table 3) is consistent with previous studies on ruminants fed VIN or similar molasses‐based by‐products (Leontowicz et al. 1994; Zali et al. 2017, 2019). This lack of impact suggests that VIN, when top‐dressed on a TMR, is palatable and does not disrupt feeding behaviour in early‐lactating Holstein cows, a period characterized by high metabolic demands and susceptibility to intake reductions (Allen 1997). Although variability in DMI responses exists in the literature, with reduced DMI at higher VIN inclusion levels in some cases (Lopez‐Campos et al. 2011; Ma et al. 2021), the consistent DMI in our study (Table 3) supports VIN's suitability as a feed additive during early lactation, where maintaining intake is critical for energy balance and milk production.

The linear increases in milk yield, FCM, ECM and FE (Table 3) indicate that VIN supplementation enhances nutrient utilization, enabling greater milk production without requiring additional feed intake. This aligns with Ma et al. (2021), who reported increased milk yield in cows fed 400 g/day VIN, though yields declined at 900 g/day, suggesting an optimal inclusion range of 300–600 g/day. In contrast, Rahimi et al. (2021) found no effect on milk yield in mid‐lactation cows fed 5%–10% VIN, likely due to lower metabolic demands compared to early lactation, where cows prioritize milk synthesis over body maintenance (NRC 2001). The quadratic response of milk fat concentration (peaking at 1.5% VIN; Table 3) and linear increases in yields of fat, true protein and lactose (Table 3) suggest that VIN improves energy and protein availability, likely driven by enhanced nutrient digestibility (Table 5) and favourable rumen fermentation (Table 6).

The linear increases in de novo and saturated FA concentrations in milk (Table 4) reflect enhanced mammary gland synthesis, driven by increased acetate availability (Table 6) and higher ruminal pH (Fukumori et al. 2021). De novo FAs (C4–C14) are synthesized from acetate and BHBA (Lizhi et al. 2014), and their increase suggests that VIN supports lipid metabolism during early lactation, a period of high energy demand. The lack of effect on mixed, preformed, unsaturated, monounsaturated and polyunsaturated FAs, as well as C16:0, C18:0 and C18:1 *cis*‐9 (Table 4), indicates that VIN primarily influences de novo synthesis rather than dietary or mobilized fat uptake. This is advantageous for maintaining milk fat quality without altering the balance of preformed FAs, which are more dependent on dietary lipids or body fat mobilization (Bach et al. 2019).

The linear increases in DM, OM and CP digestibility (Table 5) are likely attributable to VIN's unique composition, including high NPN (27.8% of CP, predominantly aspartic acid, glutamic acid and betaine; Table 2), potassium (8.8% DM) and *Saccharomyces cerevisiae* yeast residues, which may have prebiotic effects (Stemme et al. 2005; Dias et al. 2018). NPN is rapidly degraded in the rumen, providing ammonia for microbial protein synthesis (Bach et al. 2005; Hannon and Trenkle 1990), which likely contributes to increased milk protein yield (Table 3). The high potassium content elevates ruminal pH (Table 6), creating an optimal environment for *cellulolytic* bacteria and enhancing DM and CP digestion (Russell 1998; Jenkins et al. 2014). The lack of effect on NDF and ADF digestibility (Table 5) is consistent with Fernández et al. (2009), who found no improvement in fibre digestion in ewes fed 7%–13% VIN, suggesting that VIN's benefits are primarily associated with non‐fibrous components of the diet.

The linear increases in plasma globulin, total protein and BUN (Table 7), coupled with a linear decrease in glucose, require careful interpretation. Elevated BUN reflects VIN's high NPN content (Table 2), which increases rumen ammonia production, subsequently converted to urea in the liver (Allison 1970; Bach et al. 2005). This is consistent with Lopez‐Campos et al. (2011), who reported higher BUN in lambs fed VIN. The glucose decreases likely results from increased demand for lactose synthesis during higher milk production (Table 3; Lohrenz et al. 2010; Ma et al. 2021). The increase in plasma globulin, a potential marker of inflammation (Ceciliani et al. 2012; Cattaneo et al. 2021), may suggest a mild inflammatory response, possibly triggered by VIN's high potassium or NPN content (Table 2). High dietary potassium can alter electrolyte balance, potentially inducing stress in early‐lactating cows, whereas excessive NPN may challenge rumen ammonia clearance (Lopez‐Campos et al. 2011). However, without data on inflammatory markers (e.g., haptoglobin and serum amyloid A) or rumen microbial profiles, these interpretations remain speculative. The absence of changes in cholesterol, triglyceride, albumin and albumin‐to‐globulin ratio (Table 7) aligns with Moeini et al. (2014) and Zali et al. (2019), who found no effect of molasses solubles on lipid or albumin metabolism in lambs and goat kids, respectively. This suggests that VIN's metabolic effects are more pronounced in early‐lactating cows, likely due to their heightened metabolic sensitivity.

The potential benefits of VIN, including improved milk yield, FE and nutrient digestibility, suggest it could reduce feed costs and environmental waste by repurposing a sugar beet processing by‐product. However, the increased BUN and globulin levels highlight potential metabolic and immune challenges, particularly in early lactation when cows are metabolically stressed. Additionally, the high potassium content of VIN could pose risks for mineral imbalances in long‐term feeding, necessitating further evaluation of dietary cation–anion balance (Jenkins et al. 2014). Economic analyses comparing VIN to conventional feed additives (e.g., urea and molasses) are also needed to determine its cost‐effectiveness.

Although this study provides valuable insights into the effects of VIN supplementation on early lactating Holstein dairy cows, several limitations should be acknowledged. The 45‐day duration may not capture long‐term effects of VIN on cow health or productivity. The absence of microbial profiling limits insights into VIN's impact on rumen microbiota. Reproductive performance and inflammatory markers (e.g., acute‐phase proteins) were not assessed, restricting interpretation of increased plasma globulin. The small sample size (*n* = 8 per treatment) and focus on early‐lactating cows may limit generalizability. Larger, longer‐term studies are needed to validate these findings.

## 5 Conclusions

Under the conditions of this study, VIN supplementation at 1.5% or 3% of the diet did not affect DMI but linearly increased milk yield, FCM, ECM, FE and milk fat, protein, lactose and SNF yields in early‐lactation Holstein cows. Nutrient digestibility (DM, OM and CP), ruminal pH and acetate:propionate ratio improved, whereas plasma glucose decreased and globulin, total protein and BUN increased. These findings suggest VIN is a promising feed additive for enhancing performance, but elevated globulin levels warrant caution due to potential inflammatory implications. Long‐term studies are needed to assess VIN's effects on health, immune function and cost‐effectiveness before widespread adoption.

## Author Contributions

**A. Rahimi**: conceptualization, data curation, formal analysis. **F. Fatehi**: conceptualization, project administration. **H. Rafiee**: conceptualization, methodology, validation and writing – original draft, writing – review and editing. **A. Kahyani**: data curation, investigation, methodology and review and editing.

## Funding

The authors have nothing to report.

## Ethics Statement

The authors have nothing to report.

## Conflicts of Interest

The authors declare no conflicts of interest.

## Data Availability

Data are available on request.
